# Health economic analysis of costs of laparoscopic and open surgery for rectal cancer within a randomized trial (COLOR II)

**DOI:** 10.1007/s00464-016-5096-2

**Published:** 2016-07-15

**Authors:** Jacob Gehrman, Ingela Björholt, Eva Angenete, John Andersson, Jaap Bonjer, Eva Haglind

**Affiliations:** 1 Department of Surgery, Institute of Clinical Sciences, Sahlgrenska Academy, University of Gothenburg, SSORG—Scandinavian Surgical Outcomes Research Group, Sahlgrenska University Hospital/Östra, SE-416 85 Gothenburg, Sweden; 2 Nordic Health Economics, Gothenburg, Sweden; 3 Department of Surgery, VUmc University Medical Centre, Amsterdam, The Netherlands

**Keywords:** Health economics, Cost-minimization analysis, Costs, Rectal cancer surgery

## Abstract

**Background:**

Previous studies regarding the comparative costs of laparoscopic and open surgery for rectal cancer provide ambiguous conclusions, and there are no large randomized trials or long-term follow-up.

**Methods:**

A prospective cost-minimization analysis was carried out by using data of clinical resource use from the randomized controlled trial COLOR II. Some data needed for the health economic evaluation were not collected in the clinical trial; therefore, a retrospective data collection was made for COLOR II-patients operated at the largest participating Swedish hospital (*n* = 105). Sick leave information was provided by the Swedish social insurance agency. Unit costs were collected from Swedish sources. The primary outcome was the difference in mean cost between laparoscopic and open surgery.

**Results:**

The COLOR II-trial enrolled 1044 rectal cancer patients randomized between laparoscopic and open surgery 2:1. At the 3-year follow-up data for the clinical variables used in the analysis were available for 74–89 % of patients. Laparoscopic surgery costs the health care sector more than the open technique, both at 28 days ($1910, 95 % CI 677–3143) and 3 years ($3854, 95 % CI 1527–6182) after surgery. There were, however, no differences in long-term costs to society between laparoscopic and open surgery ($684, 95 % CI −5799 to 7166).

**Conclusions:**

Though the study found short- and long-term cost differences for the healthcare sector, there was no difference in regard to the long-term societal perspective. Future research is suggested to investigate the effects of sick leave costs using material from a greater number of patients.

## Background

Several smaller series and one large randomized trial, the COLOR (COlorectal cancer Laparoscopic or Open Resection) II-trial [1], have shown that laparoscopic surgery for rectal cancer has short-term benefits and is safe in comparison to open surgery. The short-term outcomes of the COLOR II-trial found that the laparoscopic group had less blood loss and a shorter hospital stay, but longer operating room time [2]. The analysis of the primary endpoint showed no difference with regard to loco-regional recurrence rates. There was no statistically significant difference in 3-year survival between the surgical procedures [3]. The study continues to monitor the disease-free and overall survival rates 5 years after surgery. The short-term outcomes of the ACOSOG Z6051 [4] and ALaCaRT [5] randomized clinical trials of laparoscopic and open rectal cancer resections failed to establish non-inferiority in terms of the pathological and adequate surgical resection outcomes. These trials have, however, used other endpoints, both are short-term and the group sizes are such that clinically relevant long-term oncological results cannot be ascertained.

Uncertainties remain regarding the relative costs of laparoscopic and open rectal cancer surgery. Several studies performed alongside randomized trials comparing the costs of laparoscopic and open surgery for rectal cancer have had short time perspectives [6–9] or have not included the cost of sick leave [8–10]; the results are difficult to interpret from a societal viewpoint.

The aim of the present study was to evaluate the cost of laparoscopic versus open resection for rectal cancer from both the healthcare and the societal perspective, based on the randomized COLOR II-trial. The health economic method employed was a cost-minimization analysis (see health economic methodology). The costs were assessed at 28 days (short-term analysis) and 3 years (long-term analysis). The hypothesis was that laparoscopic surgery would be more costly when assessed at 28 days after the primary operation but not at 3 years.

## Materials and methods

### The COLOR II-trial

The COLOR II-trial provided the clinical data for the present cost study [11]. The study was designed as a non-inferiority trial undertaken at thirty hospitals in eight countries (Belgium, Canada, Denmark, Germany, the Netherlands, Spain, Sweden and South Korea) between January 2004 and May 2010 [11]. The patients were randomized on a 2:1 basis, 699 patients in the laparoscopic resection group and 345 in the open resection group. The trial was stratified by center, location of tumor and radiotherapy prior to surgery [2, 11]. During the course of the trial, clinical record forms (CRF) were administered, one each for the pre-, intra- and postoperative stages (up to 28 days after the operation) and one CRF per year up to 5 years after the index surgery. In case of complications, reoperations or recurrences an additional CRF was completed. At the primary endpoint data were available for 771 patients (74 %) regarding loco-regional recurrence and for 903 patients (87 %) concerning overall survival [3]. The institutional review board at each participating center approved the trial. All patients provided informed consent in writing.

### Health economic methodology

Health economic evaluations such as cost-effectiveness analysis are based on the incremental cost for an incremental unit of a clinically relevant outcome (mortality or morbidity) or a QALY (quality-adjusted life-years) as a measure of treatment or program effectiveness [12]. Survival and health-related quality of life [13, 14] were not statistically different in COLOR II (N.B. non-inferiority trial), and consequently a cost-minimization rather than a cost-effectiveness analysis was appropriate for the analysis [15]. This method implies a comparison of the costs for treatment alternatives that achieve a common outcome to an equal degree [12]. The rationale for the included cost components in the present study is outlined in more detail in Björholt et al. [16]

The cost analysis comprises the health care and the societal perspective, where the latter adds the cost of sick leave to the direct healthcare cost. The study period was set from inclusion into the clinical trial up to 3 years postoperatively, including the short- and long-term clinical endpoints of the COLOR II-study. Censoring and missing data can cause bias in economic studies conducted alongside clinical trials [17]. In this trial, the return rate of clinical record forms was high and it was assumed from a clinical perspective that censored patients and patients with missing data would not differ from non-censored patients and patients with complete data in the aspects affecting cost. One-way sensitivity analysis was employed to challenge the impact of variables sensitive to censoring mechanisms and missing data, i.e., reoperation, stoma care and sick leave. The analysis shows how the difference in mean cost between the surgical techniques is affected by changes (±30 %) in cost per variable for each procedure.

### Data collection

#### Resource use

Data on resource use were collected prospectively through CRF’s in the COLOR II-trial. Details of the use of resources that were needed for this study, but had not been collected within the trial (basic laparoscopic equipment, surgical instruments, anesthesia time and time in recovery room), were determined using other sources.

The basic equipment required for laparoscopic surgery, as well as the type and quantity of instruments required for laparoscopic and open surgery, was determined by conclusions drawn from the health economic evaluation of laparoscopic versus open colon cancer resection within the framework of the randomized trial COLOR [18] and in collaboration with senior surgeons. Data regarding duration of anesthesia and time in the recovery room were collected from the records of COLOR II-patients operated on at the Sahlgrenska University Hospital in Sweden (*n* = 105). The factor between time in anesthesia and skin-to-skin time was established for the Sahlgrenska patients and applied on all COLOR II-patients. The average time in the recovery room for COLOR II-patients operated on at Sahlgrenska University Hospital was extrapolated to all study participants.

Sick leave was retrieved from the Swedish Social Insurance Agency for Swedish COLOR II-patients, and the observed average number of days on sick leave per surgical technique was calculated. To be able to analyze the total cost at the patient level, the average number of sick leave days per surgical technique observed in the Swedish cohort was applied to the non-Swedish COLOR II-population using random selection. It was ascertained that the proportion of patients on sick leave in the Swedish cohort, and the non-Swedish COLOR II-populations was the same.

#### Unit costs

Unit costs for basic equipment and surgical instruments were obtained from regional procurement records in Region Västra Götaland, Sweden. The cost per minute in the operating room, time in anesthesia and time in the recovery room were derived from the health economic evaluation of laparoscopic versus open surgery in the COLOR trial [18]. The unit costs for consumables related to stoma care were obtained from pharmacy retail prices in Sweden. The Swedish cost per patient database contains cost data for approximately 70 % of inpatient care in Sweden. The unit cost per type of reoperation in this study was estimated by taking the average cost for the matching procedure in the cost per patient database. Therefore, the cost per reoperation was based on a larger sample which reduced the potential variability in resource use of these rare and costly events. It was assumed that the type of reoperation was unrelated to the original surgical technique, as no significant differences in complications or re-operations were found in the COLOR II-study [2].

The cost of sick leave was calculated by using the average monthly wage (provided by Statistics Sweden) with addition of the social security and supplementary pension fees. All prices were inflation adjusted for 2013 SEK using the consumer price index provided by Statistics Sweden. Costs were converted from SEK to the average value of the US dollar in 2013 ($1 = 6.51 SEK).

### Statistical methods

The distribution of cost data is non-negative and right skewed due to the low number of patients incurring particularly high costs, a common phenomenon in studies involving resource items with high unit costs such as hospital care, reoperations and sick leave. The average cost will consequently be higher than the cost of the average patient, but it is still meaningful as it enables the calculation of the total cost of treating all patients with the new therapy [17]. Due, however, to the central limit theorem statistical inference based on the normality assumption regarding average cost is valid despite the skewed distribution. A non-parametric bootstrap was included as a robustness check of the results [19].

## Results

**Fig. 1:**
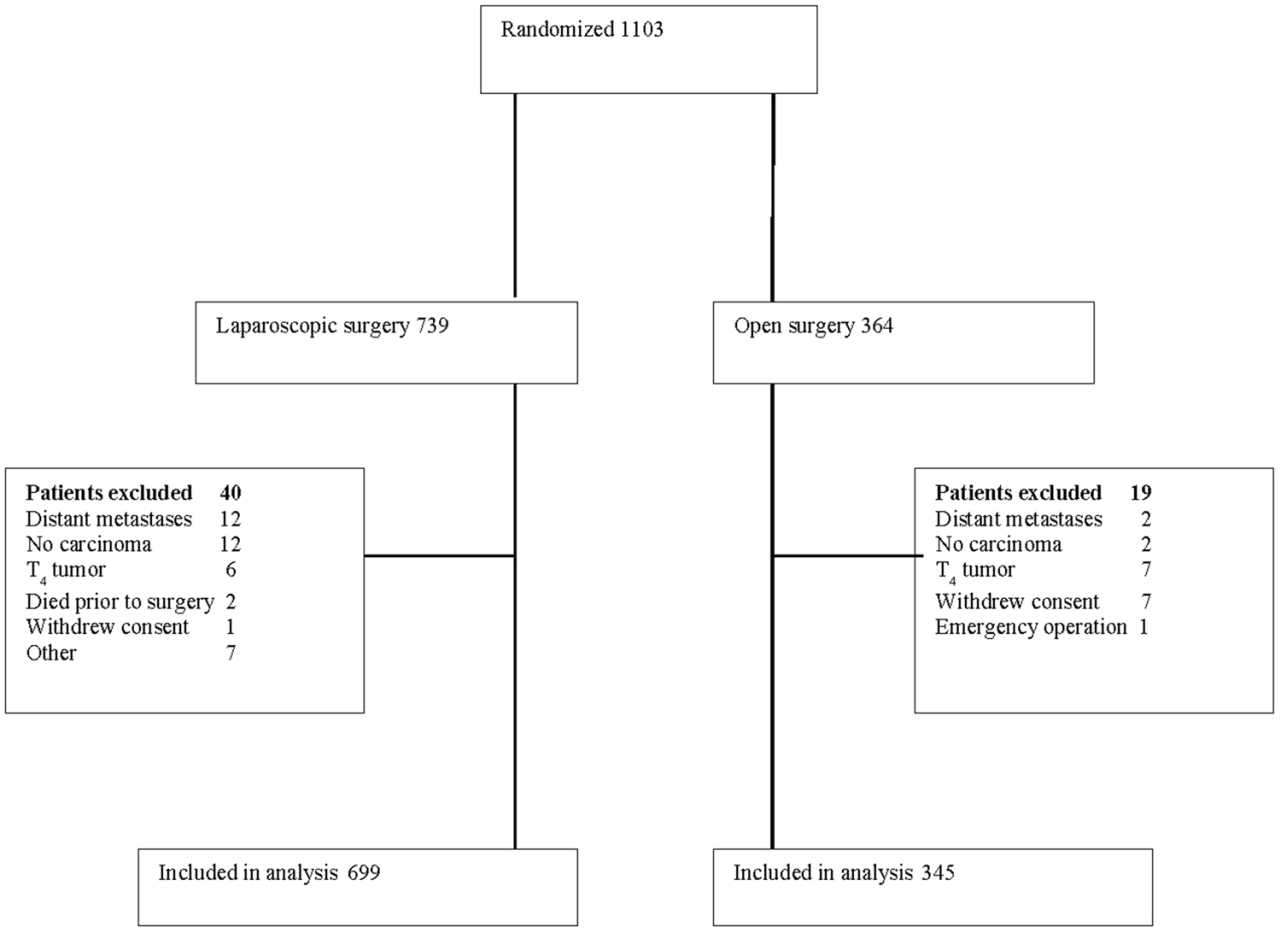
Study flow chart and COLOR II

For the health economic study, 699 patients in the laparoscopic group and 345 in the open group were available for analysis (Fig. 1). Information relevant for the short-term outcomes of the study was available for between 98 and 100 % of the patients. Concerning the long-term analysis data were available for 74–89 % of the patients. The baseline clinical characteristics (Table 1) were not significantly different between laparoscopic and open resection. The resource use and the corresponding unit costs associated with each treatment are shown in Tables 2 and 3, respectively. Table 4 displays the mean cost per resource use category and treatment, and Table 5 shows the difference in means and the main outcome of the study. The bootstrap method did not affect the *p* value for any of the results in Table 5, but the confidence intervals became narrower.

**Table 1:** Baseline clinical characteristics and pathology

Characteristics	Laparoscopic surgery (*n* = 699)	Open surgery (*n* = 345)
Gender, no. (%)		
Male	448/699 (64)	211/345 (61)
Female	251/699 (36)	134/345 (39)
Age, mean (SD), years	66.8 (10.5)	65.8 (10.9)
American Society of Anesthesiologists classification, no. (%)		
I	156/678 (23)	65/338 (19)
II	386/678 (57)	211/338 (62)
III	131/678 (19)	61/338 (18)
IV	5/678 (<1)	1/338 (<1)
Missing data	21/699 (3)	7/345 (2)
Body-mass index, mean (SD), kg/m^2^	26.1 (4.5)	26.5 (4.7)
Location of tumor (distance from anal verge), no. (%)		
Upper rectum (10–15 cm)	223/699 (32 %)	116/345 (34)
Middle rectum (5–10 cm)	273/699 (39 %)	136/345 (39)
Lower rectum (<5 cm)	203/699 (29 %)	93/345 (27)
Clinical stage, no. (%)		
I	201/667 (30)	96/329 (29)
II	209/667 (31)	107/329 (33)
III	257/667 (38)	126/329 (38)
Missing data	32/699 (5)	16/345 (5)

**Table 2:** Clinical resource use

Type of resource	Laparoscopic surgery (*n* = 699)	Open surgery (*n* = 345)	Source
Basic laparoscopic equipment, no (%)	699 (100)	0 (0)	COLOR II
Surgical instruments, no (%)^a^			COLOR II
TME	396/699 (57)	219/345 (63)	
Other (APR, PME)	294/699 (42)	126/345 (37)	
Missing	9/699 (1)	0/345 (0)	
Skin-to-skin time, min	247 (83)	200 (69)	COLOR II
Time in anesthesia, min^b^	306 (104)	256 (89)	Subset of COLOR II-patients
Time in recovery room, min^b,c^	992 (N/A)	1054 (N/A)	Subset of COLOR II- patients
Length of hospital stay < 28 days, days	11.5 (6.5)	12.1 (6.0)	COLOR II
Length of hospital stay < 3 years, days	12.8 (11.5)	13.4 (11.0)	COLOR II
Days with ileostomy < 28 days, days	10.5 (13.5)	10.8 (13.6)	COLOR II
Days with ileostomy < 3 years, days	91 (182)	80 (139)	COLOR II
Days with colostomy < 28 days, days	9.7 (13.2)	8.1 (12.6)	COLOR II
Days with colostomy < 3 years, days	363 (502)	281 (461)	COLOR II
No. (%) of patients with reoperation < 28 days			COLOR II
No	588/697 (84)	299/345 (87)	
Yes	109/697 (16)	46/345 (13)	
No. (%) of patients with reoperation < 3 years			COLOR II
No	459/697 (66)	240/345 (70)	
Yes	238/697 (34)	105/345 (30)	
Reasons for reoperation (< 3 years)^d^			COLOR II
Recurrence	28	14	
Complication	217	89	
Stoma reversal	68	28	
Complication and new stoma	48	21	
Not related to rectal surgery	10	7	
Sick leave < 28 days, days^e^	6.2 (11.6)	6.4 (11.8)	Swedish Social Insurance Agency
Sick leave < 3 years, days^e^	75 (137)	86 (157)	Swedish Social Insurance Agency

Values are mean (standard deviation) unless stated otherwise

^a^TME-Resection with total mesorectal excision, APR-Abdominoperineal resection, PME-Resection with partial mesorectal excision. Described in more detail in van der Pas et al. [2]. A set of surgical instruments used for open and laparoscopic TME and non-TME was determined in collaboration with senior surgeons. The number of TME and non-TME was collected from the COLOR II-trial

^b^Collected within the Swedish cohort of COLOR II operated at the Sahlgrenska University Hospital, Gothenburg

^c^The mean value of time in recovery room in the Swedish cohort and in the global study population all patients were assigned these mean values, i.e., std. dev. not possible to calculate

^d^Long-term data (< 3 years) from RCT COLOR II previously not published. Several patients have had more than one reoperation collected within the Swedish cohort of COLOR II only

^e^Collected within the Swedish cohort of COLOR II only

**Table 3:** Unit costs

Resource use category	Unit cost (USD)	Unit	Source
Basic laparoscopic equipment	281	Per laparoscopic resection	Region Västra Götaland, Sweden
Surgical instruments^a^			Region Västra Götaland, Sweden
Open TME	931	Per open TME	
Laparoscopic TME	2101	Per laparoscopic TME	
Open non-TME	784	Per open non-TME	
Laparoscopic non-TME	1779	Per laparoscopic non-TME	
Skin-to-skin time	11	Per minute	Janson et al. 2004
Duration of anesthesia	5	Per minute	Janson et al. 2004
Time in recovery room	1	Per minute	Janson et al. 2004
Length of hospital stay	531	Per day	Janson et al. 2004
Ileostomy	12	Per day	Pharmacy sales price
Colostomy	18	Per day	Pharmacy sales price
Reoperation^b^	N/A	Per type of reoperation	Swedish association of local authorities and regions
Sick leave	303	Per day	Statistics Sweden

^a^TME-Resection with total mesorectal excision, APR-Abdominoperineal resection and PME-Resection with partial mesorectal excision. Described in more detail in van der Pas et al. [2]

^b^The type of reoperation was collected within the COLOR II-trial. The unit cost per type of reoperation was collected from a national database (Swedish association of local authorities and regions) containing the costs for approximately 70 % of the Swedish in-patient episodes of care

**Table 4:** Mean cost and difference in mean cost per resource use category

Resource use category	Mean cost per patient/laparoscopic surgery (USD)	Mean cost per patient/open surgery (USD)	Difference in mean costs (USD)
Basic laparoscopic equipment	281 (0)	N/A	281 (N/A)
Surgical instruments	1964 (159)	878 (71)	1087 (7)
Skin-to-skin time	2676 (898)	2161 (750)	514 (53)
Duration of anesthesia	1545 (526)	1293 (451)	252 (32)
Time in recovery room	1074 (N/A)	1141 (N/A)	−67 (N/A)
Length of hospital stay			
28 days	6129 (3427)	6431 (3210)	−302 (221)
3 years	6796 (6129)	7117 (5844)	−321 (398)
Stoma			
Ileostomy 28 days	124 (158)	127 (160)	−3,6 (10)
Ileostomy 3 years	1070 (2142)	941 (1630)	129 (120)
Colostomy 28 days	174 (238)	146 (227)	27 (15)
Colostomy 3 years	6519 (9022)	5038 (8288)	1481 (578)
Reoperation			
28 days	2397 (7280)	2199 (7591)	198 (486)
3 years	5902 (11,867)	5323 (11,580)	578 (775)
Sick leave			
28 days	1888 (3447)	1945 (3575)	−58 (230)
3 years	22,793 (41,618)	25,964 (47,712)	−3171 (3014)

Values are mean (standard deviation), except difference in mean costs (standard error)

**Table 5:** Mean total cost and difference in mean total cost per surgical technique

Perspective and time of analysis	Mean total cost laparoscopic resection (USD)	Mean total cost open resection (USD)	Difference in mean total cost (95 % CI)	*p* value
Health care costs 28 days				
Parametric	16,226 (10,140)	14,316 (10,361)	1910 (677 to 3143)	<0.002
Nonparametric (bootstrap)			1910 (685 to 3123)	<0.003
Health care costs 3 years				
Parametric	27,686 (46,198)	23,831 (51,993)	3854 (1527 to 6182)	<0.001
Nonparametric (bootstrap)			3854 (1491 to 6053)	<0.001
Societal costs 28 days				
Parametric	18,113 (9524)	16,261 (9591)	1852 (533 to 3171)	<0.006
Nonparametric (bootstrap)			1852 (391 to 3110)	<0.006
Societal costs 3 years				
Parametric	50,479 (18,162)	49,795 (17,719)	684 (−5799 to 7166)	0.84
Nonparametric (bootstrap)			684 (−5698 to 7255)	0.84

Values are mean (standard deviation) unless stated otherwise. Bootstrap confidence intervals and *p* values are based on 2000 replications

### Healthcare perspective

The mean healthcare cost per patient (Table 5) during the 28 days following surgery was significantly higher in the laparoscopic group $16226 (SEK, 105694) than in the open group $14316 (SEK, 93253), yielding a difference of $1910 (SEK, 12440) (CI95 % 677–3143). Three years after surgery, this difference had increased to $3854 (SEK 25107) (CI95 % 1527–6182).

### Societal perspective

From the societal perspective, the mean cost per patient (Table 5) at 28 days following surgery was significantly higher in the laparoscopic group $18113 (SEK, 117990) than in the open group $16261 (SEK 105926), with a difference of $1852 (SEK, 12063) (95 % CI 533–3171). Three years after surgery, the difference was not significant and had decreased to $684 (SEK, 4453) (95 % CI −5799 to 7166).

### Sensitivity analyses

Sensitivity analyses can be found in Table 6. From the short-term healthcare and societal perspectives length of hospital stay were the only variable demonstrating significant sensitivity (difference in mean cost became negative) to the variation of the base case value. Long-term societal costs were affected by the number of days on sick leave.

**Table 6:** Sensitivity analyses

Perspective and time of analysis	Variable	Change in cost	Difference in mean (USD)	95 % CI	
Health care costs 28 days					
	Base case cost	N/A	1910	677	3143
	Skin-to-skin time	Lap −30 %	1103	−125	2330
		Lap +30 %	2671	1430	3912
		Open −30 %	2524	1293	3755
		Open +30 %	1250	13	2487
	Length of hospital stay	Lap −30 %	79	−1097	1255
		Lap +30 %	3740	2444	5037
		Open −30 %	3839	2632	5046
		Open +30 %	−19	−1282	1243
	Colostomy	Lap −30 %	5970	2671	9269
		Lap +30 %	1700	−1708	5108
		Open −30 %	1853	−641	4348
		Open +30 %	1927	−568	4422
	Reoperation	Lap −30 %	1191	31	2350
		Lap +30 %	2629	1299	3958
		Open −30 %	2570	1505	3634
		Open +30 %	1250	−180	2679
Health care costs 3 years					
	Base case cost	N/A	3854	1527	6182
	Length of hospital stay	Lap −30 %	1824	−413	4061
		Lap +30 %	5884	3455	8314
		Open −30 %	5989	3706	8272
		Open +30 %	1719	−658	4097
	Reoperation	Lap −30 %	2084	−115	4283
		Lap +30 %	5625	3181	8068
		Open −30 %	5451	3365	7538
		Open +30 %	2257	−308	4823
Societal costs 28 days					
	Base case cost	N/A	1852	533	3171
	Surgical instruments	Lap −30 %	1269	−50	2588
		Lap +30 %	2435	1115	3755
		Open −30 %	2115	796	3434
		Open +30 %	1589	269	2908
	Skin-to-skin time	Lap −30 %	1124	−102	2351
		Lap +30 %	2695	1455	3935
		Open −30 %	2547	1316	3777
		Open +30 %	1273	37	2509
	Length of hospital stay	Lap −30 %	21	−1244	1286
		Lap +30 %	3683	2302	5063
		Open −30 %	3781	2486	5076
		Open +30 %	−77	−1424	1270
	Reoperation	Lap −30 %	1133	−126	2391
		Lap +30 %	2571	1155	3987
		Open −30 %	2512	1344	3679
		Open +30 %	1192	−321	2705
	Sick leave	Lap −30 %	1286	−7	2579
		Lap +30 %	2418	1064	3772
		Open −30 %	2436	1132	3740
		Open +30 %	1268	−71	2607
Societal costs 3 years					
	Base case cost	N/A	684	−5799	7166
	Skin-to-skin time	Lap −30 %	3069	749	5389
		Lap +30 %	4640	2305	6974
		Open −30 %	4491	2189	6794
		Open +30 %	3217	887	5548
	Sick leave	Lap −30 %	−6154	−12,241	−67
		Lap +30 %	7522	541	14,502
		Open −30 %	8473	3121	13,825
		Open +30 %	−7106	−14,852	641

*Lap* Laparoscopic resection, *open* open resection

### Additional sensitivity analysis

The incidence of colostomy had considerable impact on the study result and so did the number of days on sick leave. A detailed analysis of the data showed that patients in the open surgery group were older, and therefore, fewer were eligible for sick leave compared with patients in the laparoscopic group. Numerically more patients on sick leave in the laparoscopic group died earlier compared to the open group which resulted in a lower cost of sick leave in the laparoscopic group. As survival did not significantly differ between the groups, this was most likely a random finding due to sick leave only having been examined in a small sub-group of the COLOR II-trial. The mean number of days with a colostomy was higher in the laparoscopic group, partly due to numerically more laparoscopic patients subject to abdominoperineal resection at the index operation and partly due to longer survival time in the sub-group of colostomy patients in the laparoscopic group. Additional sensitivity analyses were, therefore, performed and the results showed that excluding the costs of stoma material the difference in mean cost per patient to the health care sector ($1886, 95 % CI 657–3115) (SEK, 12286) was similar to the base case analysis at 28 days after the index surgery. At 3 years the difference in mean health care cost per patient ($2245, 95 % CI 270–4219) (SEK, 14621) was lower compared to the base case analysis ($3854). Laparoscopic surgery was numerically less costly per patient for society than open surgery −$926 (SEK, −6034) (95 % CI −7261 to 5409) at 3 years after primary operation. In the short time perspective, it made little difference ($1828, 95 % CI 513–3144) (SEK, 11909).

## Discussion

This health economic evaluation of laparoscopic and open surgery for rectal cancer in the framework of the randomized trial COLOR II showed that laparoscopy was significantly more costly from the societal perspective at 28 days, but no statistical significance was detected at 3 years. From a healthcare perspective, laparoscopy was more costly than open surgery at both 28 days and 3 years.

The one-way sensitivity analyses showed that variations in sick leave and the incidence and days with colostomy had a large impact on the results. Data on sick leave were elicited for the Swedish sub-group only (*n* = 251), which increased the risk of random findings. This is a common problem for costly resource use items that may vary without relation to the studied interventions. In this study, the finding was a disadvantage to the results in the open surgery group. On the other hand, patients with a cancer in the lowest part of the rectum, who received a colostomy due to abdominoperineal resection, lived longer (n.s.) and were numerically more frequent in the laparoscopic group [3]. Since colostomies are costly and a longer follow-up time involves further costs, this was a disadvantage for the laparoscopic group. The additional sensitivity analyses confirmed that when stoma costs were deducted from the health care costs, the difference in mean cost per patient was reduced. For the long-term societal costs, the difference (laparoscopic minus open surgery) in mean cost per patient changed from $684 (base case) to -$926 (n.s.).

There are few findings about the cost of rectal cancer surgery and they are divergent. Franks et al. [6] reported on a randomized trial including the initial 3 months after index surgery and found no significant difference in societal costs between open and laparoscopic surgery, but the time chosen for their analysis differs from that of our analysis and the number of patients were fewer in their study. Son et al. [8] found statistically significant higher median costs for laparoscopic rectal resection compared to open, utilizing data for a cohort of a randomized trial. Using median costs makes it difficult to compare it to the results of the present study, since our results present mean costs. Their health economic evaluation covered the first three postoperative months and healthcare costs only, whereas our analysis covers 3 years and includes societal costs.

The results from the societal perspective in this trial correspond to those from the earlier trial of laparoscopic versus open surgery for colon cancer (COLOR) [18], although the time frame was shorter in that study. Three previous studies have reported the cost of sick leave after colorectal cancer. In Franks et al. [6], the average cost of sick leave after rectal cancer surgery was higher in the laparoscopic than in the open resection group (the difference in average cost was £103 95 % CI £−576 to £368). King et al. [7] reported the difference in average cost of productivity loss between laparoscopic and open resection of colorectal cancer within an enhanced recovery program to be £274 (bootstrap CI at 2.5 and 97.5 %, −386 to 983.2) less in the laparoscopic resection group. In a recent study by Crawshaw et al. [20], the difference in sick leave after colectomy was estimated to be on average 2.78 (95 % CI 1.93–3.59) days longer in the open resection group than in the laparoscopic resection group. That study was retrospective and based on national health insurance claims in USA. They evaluated health care utilization up to 1 year after primary operation and found the mean cost to be lower following laparoscopic surgery. Our study has a longer time perspective (3 years) and is based on a randomized controlled trial.

In the present study, the length of hospital stay was considerable in both groups, although 1 day shorter (median) in the laparoscopic group. The trial protocol prescribed that the same local principles for discharge should be applied for both groups and did not include the enhanced recovery after surgery (ERAS) concept [21]. The difference in hospital stay, however, between laparoscopic and open colorectal surgery is consistent with studies including enhanced recovery programs [22, 23]. The sensitivity analyses conducted in the present study indicated a potential for cost saving if length of hospital stay can be shortened. In one study, King et al. [7] evaluated the costs following laparoscopic and open surgery after colorectal cancer surgery within an enhanced recovery program and found a difference in mean cost of £354 (95 % CI −2 167 to 2 992) favoring laparoscopic surgery.

The strengths of our study include that it is based on clinical results from a large randomized trial with a multicenter design and that the principles for the health economic analysis were outlined before the start of the randomized trial. Thus, the clinical record forms included variables of importance for the economic analysis. The study also had a high rate of returned clinical record forms.

A limitation is that the present health economic analysis was a secondary objective within the randomized trial COLOR II and the sample size, thus, not calculated for the health economic outcomes. Further, some of the resource units are for a sub-group of patients of the COLOR II-trial, which adds to the uncertainty of these variables.

In conclusion, laparoscopic surgery for rectal cancer is more costly than open surgery from the health care perspective. It is important, however, that the cost of sick leave is taken into account to ensure inclusion of all costs arising as a consequence of the surgical method chosen. In the present study, sick leave was investigated in the Swedish cohort only which was too small to detect a true difference between the treatments. Future research is suggested to investigate the sick leave costs of rectal cancer surgery.
